# A Functional Study Identifying Critical Residues Involving Metal Transport Activity and Selectivity in Natural Resistance-Associated Macrophage Protein 3 in *Arabidopsis thaliana*

**DOI:** 10.3390/ijms19051430

**Published:** 2018-05-10

**Authors:** Jiyu Li, Lihua Wang, Lu Zheng, Yuerong Wang, Xi Chen, Wei Zhang

**Affiliations:** Department of Biochemistry & Molecular Biology, College of Life Sciences, Nanjing Agricultural University, Nanjing 210095, China; 2015216033@njau.edu.cn (J.L.); lijiyugx@163.com (L.W.); 2016116107@njau.edu.cn (L.Z.); 2017116124@njau.edu.cn (Y.W.)

**Keywords:** natural resistance-associated macrophage protein, *Arabidopsis thaliana*, tonoplast, site-directed mutagenesis, structure modeling

## Abstract

*Arabidopsis thaliana* natural resistance-associated macrophage protein 3 (AtNRAMP3) is involved in the transport of cadmium (Cd), iron (Fe), and manganese (Mn). Here, we present a structure-function analysis of AtNRAMP3 based on site-directed mutagenesis and metal toxicity growth assays involving yeast mutants, combined with three-dimensional (3D) structure modeling based on the crystal structure of the *Eremococcus coleocola* NRAMP family transporter, EcoDMT. We demonstrated that two conservative sites, D72 and N75, are essential for the transport activity. The M248A mutation resulted in a decrease in Cd sensitivity, while maintaining Mn transport. The mutation involving G61 caused a significant impairment of Fe and Mn transport, thereby indicating the importance of the conserved residue for proper protein function. The mutation involving G171 disrupted Fe transport activity but not that of Mn and Cd, suggesting that G171 is essential to metal binding and selectivity. Two residues, E194 and R262, may play an important role in stabilizing outward-facing conformation, which is essential for transport activity. Deletion assays indicated that the N-terminus is necessary for the function of AtNRAMP3. The findings of the present study revealed the structure-function relationship of AtNRAMP3 and metal transport activity and selectivity, which may possibly be applied to other plant NRAMP proteins.

## 1. Introduction

Transition metal ions serve as cofactors of various biological processes. Deficiency or toxicity of metal ions could inhibit plant growth and development [1]. Plants have developed different mechanisms to absorb, transport, and maintain essential metals such as iron (Fe), zinc (Zn), manganese (Mn), and copper (Cu) via protein transporters. These transporters are classified into different families, which include cation diffusion facilitator (CDF) transporters, P_1B_-type heavy metal ATPases (HMAs), yellow stripe-like (YSL) proteins, ZRT/IRT-like protein (ZIP), and natural resistance-associated macrophage protein (NRAMP).

NRAMP metal transporters have been identified in animals, bacteria, fungi, and plants. In vertebrates, the transport of free iron into the cytoplasm is catalyzed by members of the NRAMP family [2,3]. NRAMP1 constitutes a first line of intracellular defense against pathogens infection by restricting microbial access to essential micro-nutrients such as iron and manganese within professional phagosomes in humans [4]. In *E. coli*, MntH was found to be homologous to mammalian NRAMP genes and was selectively facilitated the uptake of Mn and Fe [5,6]. In rice, seven genes encode members of the NRAMP transporter family, of which four have been localized to the plasma membrane and functionally characterized, for example, OsNRAMP1 can rescue Fe uptake and enhance the accumulation of As and cadmium (Cd) when expressed in yeast [7]. Overexpression of *OsNRAMP1* in rice increases Cd accumulation in the shoots [8]. OsNRAMP3 is constitutively expressed in the nodes and is responsible for the distribution of Mn in vivo to adapt to environmental changes in Mn [9]. OsNrat1 (OsNRAMP4) transports Al^3+^ but not other divalent ions and contributes to Al detoxification [10]. OsNRAMP5 functions as a major transporter of Fe, Mn, and Cd uptake in rice [11,12,13]. In *Arabidopsis thaliana*, AtNRAMP1 functions as a high-affinity Mn transporter [14]. Loss of AtPH1 function leads to the mislocalization of AtNRAMP1 from the plasma membrane to the vacuole [15]. AtNRAMP1 also participates in the control of Fe homoeostasis in plants and interacts with *IRT1* to transport Fe in the roots under sufficient metal conditions [16,17]. Furthermore, AtNRAMP1 expression increases Cd sensitivity and accumulation in yeast [18]. AtNRAMP3 and AtNRAMP4 have been localized to the tonoplast and were involved in the transport of Cd, Fe, and Mn [18,19]. They play roles in the release of metals from vacuoles during seed germination and in the export of vacuolar Mn in the photosynthetic tissues of adult plants [20,21,22,23]. The *nramp3nramp4* double knockout mutant displays elevated sensitivity to Cd, which export much less Cd from the vacuoles to the cytosol [22]. AtNRAMP6 is an intracellular Cd transporter [24].

Recently, the crystal structures of several members of the NRAMP family members have been determined [25,26,27]. For example, *Staphylococcus capitis* DMT (*ScaDMT*), a prokaryotic homolog that transports various transition metals but not alkaline earth metal ions. The structure of the full-length protein was not clear as a shorter construct was used in crystallization, removing 41 amino acids from the N-terminus and 17 residues of the first predicted transmembrane helix at a resolution of 3.1 Å. *ScaDMT* contains 11 transmembrane helices and a single ion-binding site that is accessible from the cytoplasm. Three conserved residues (D49, N52, and M226) and a backbone carbonyl of A223 constitute the ion-binding site [26]. Soon after, the truncated structure (full length except for 13 residues on the N-terminus and five residues on the C-terminus) and functional properties of another SLC11/NRAMP transporter, *Eremococcus coleocola* (EcoDMT) was reported [27]. EcoDMT1 was crystalized in an outward facing conformation that differed from ScaDMT1 crystals which revealed the structure of an inward facing state. Another member is *Deinococcus radiodurans* Nramp (*DraNramp*), similar to *ScaDMT*, *DraNramp* comprised of 11 transmembrane helices, including TM1a, which is truncated in ScaNramp. The metal-binding site is conserved in *DraNramp* and is exposed to the extracellular side in the 3D structure obtained [25].

Mutations involving ion-coordinating residues in *ScaDMT* and *EcoDMT* result in a significant reduction in transport activity [26,27]. In contrast, methionine in *DraNramp* is not required for transition metals but influences metal selectivity [28]. Several studies have implicated the G185R mutation in anemia in humans and rodents [29,30,31,32]. Similarly, a glycine to arginine mutation (G153R) perturbs the closing of the outward metal permeation pathway and alters the selectivity of the conserved metal-binding site in *DraNRAMP* [25]. Recently, using random mutagenesis, Pottier et al. identified three mutations in *AtNRAMP4* that reduced Cd sensitivity while maintaining the ability to transport Fe [33].

Until now, studies on the structure-function relationships of members of the NRAMP family in plants are limited. Here, we used site-directed mutagenesis to investigate the structural basis of metal transport activity and selectivity in *AtNRAMP3.*

## 2. Results

### 2.1. Metal Selectivity of AtNRAMP3

**Figure 1:**
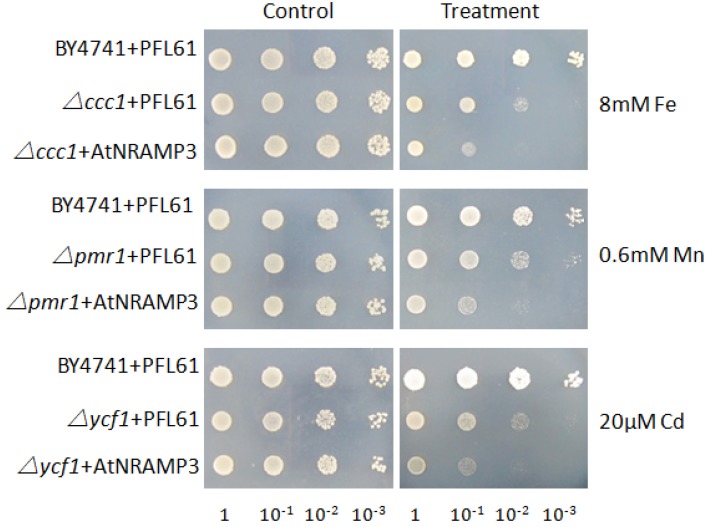
AtNRAMP3 is an Mn, Fe and Cd transporter. Wild-type and mutant yeast strains containing the empty vector or AtNRAMP3 were spotted onto synthetic dropout (SD)-Ura plates with metal supplementation as indicated.

The full-length *AtNRAMP3* cDNA (AF202539) was cloned by RT-PCR from the *Arabidopsis* sp. cDNA library. To examine the ion transport activity of AtNRAMP3, we conducted metal toxicity growth assays using yeast mutants that are sensitive to Fe (*ccc1*), Mn (*pmr1*), and Cd (*ycf1*). The results showed that AtNRAMP3 expression increased Fe, Mn, and Cd sensitivity (Figure 1), indicating that AtNRAMP3, in agreement with previous reports [18], might be a Fe^2+^, Mn^2+^ and Cd^2+^ transporter.

### 2.2. Sequence Analysis of AtNRAMP3 and Selection of Sites for Mutagenesis

**Figure 2:**
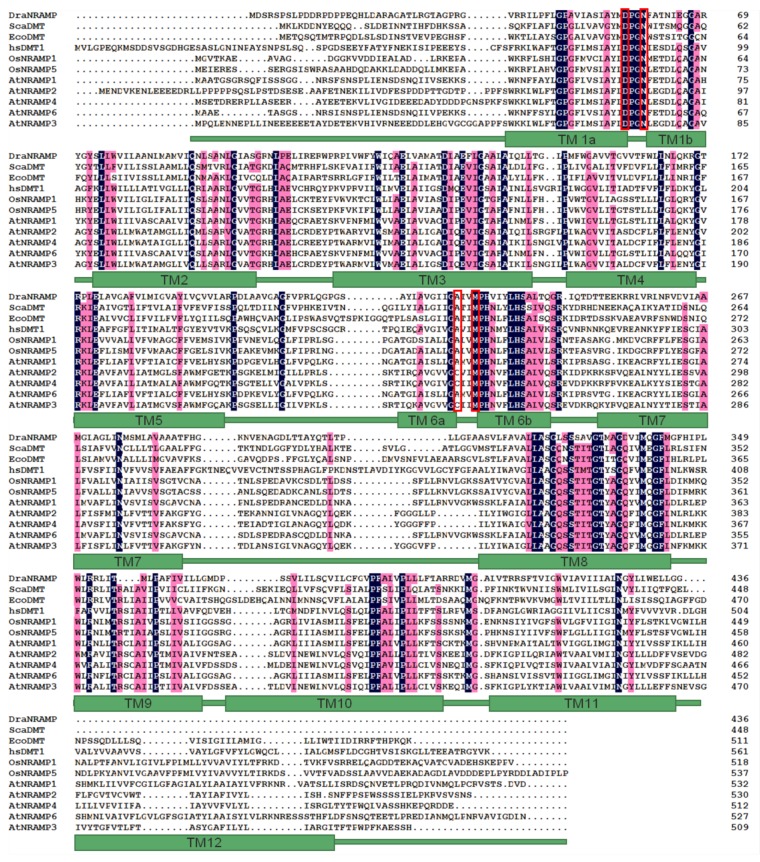
Amino acid sequence alignment of AtNRAMP3 with other NRAMP proteins. AtNRAMP1 (GenBank Acc. No. At1g80830), AtNRAMP2 (GenBank Acc. No. AT1G47240), AtNRAMP3 (GenBank Acc. No. AT2G23150), AtNRAMP4 (GenBank Acc. No. AT5G67330), and AtNRAMP6 (GenBank Acc. No. At1g15960) from *Arabidopsis thaliana*, OsNRAMP1 (Phytozome: LOC_ Os07g15460) and OsNRAMP5 (Phytozome: LOC_ Os07g15370) from *Oryza sativa*, *Staphylococcus capitis* ScaDMT (UniProtKB identifier A0A178L6Y2-1), *Deinococcus radiodurans* DraNRAMP (UniProtKB identifier Q9RTP8*), Eremococcus coleocola* EcoDMT (UniProtKB identifier E4KPW4) and human DMT1 (UniProtKB identifier P49281-2) were aligned. Identical residues are highlighted in mazarine, similar residues in pink. The putative transmembrane regions of the AtNRAMP3 were predicted based on the alignment with EcoDMT and the sequences are shown below. Selected residues of ion-binding site are indicated within a red frame.

*AtNRAMP3* encodes a putative protein of 509 amino acids in length, which was predicted to comprise 12 transmembrane domains (TMDs), based on the structure of its bacterial homolog, EcoDMT [26]. To determine which amino acid residues were likely to be required for transport activity or ion selectivity, we aligned AtNRAMP3 and other plant NRAMP transporters, including AtNRAMP1, AtNRAMP2, AtNRAMP4, AtNRAMP5, AtNRAMP6, OsNRAMP1 and OsNRAMP5 with ScaDMT (Figure 2). Multiple sequence alignment determined that 56 amino acids were conserved, which were then selected for replacement with the aliphatic amino acid residue, Ala.

### 2.3. Effects of Amino Acid Substitutions on Fe Transport Activity

In the yeast *Saccharomyces cerevisiae*, CCC1 is an Fe transporter that is responsible for transporting Fe from the cytoplasm to the vacuole. *ccc1* mutants show increased sensitivity to excessive amounts of Fe. Fe sensitivity is exacerbated by the ectopic expression of Fe transporter, FET4 [34]. Similarly, when expressed heterologously in Δ*ccc1*, AtNRAMP3 also exacerbated excessive Fe sensitivity, yeast strain *ccc1* expressing *AtNRAMP3* did not grow on a culture medium supplemented with 8 mM Fe, whereas cells transformed with an empty vector (PFL61) exhibited normal growth in the same conditions (Figure 1). Therefore, cells were transformed with plasmids carrying mutant variant genes to investigate the effect of Fe transport activity when compared to that in the wild-type AtNRAMP3 protein.

**Figure 3:**
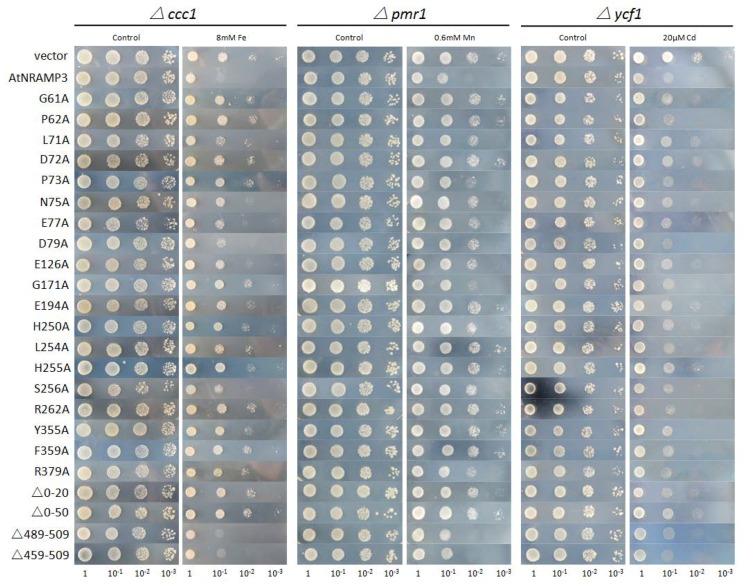
Metal toxicity growth assays of mutated AtNRAMP3 variants in *S. cerevisiae*. Yeast cell suspensions of *S. cerevisiae* mutants Δ*ccc1*, Δ*pmr1* and Δ*ycf1* transformed with empty vector PFL61, or with PFL61 containing wild-type AtNRAMP3 or mutated AtNRAMP3 cDNAs were grown on selective media with metal supplementation, respectively. For metal toxicity growth testing, transformants were pre-cultured in synthetic dropout (SD)-Ura overnight. Pre-cultured cells were diluted to an OD_600_ of 1.0, 10 μL of each cell suspension was spotted onto the selective SD-Ura plates as indicated. The plates were incubated for 72 h at 30 °C.

A total of 13 single-substitution mutations, including G61A, P62A, D72A, P73A, N75A, G171A, E194A, H250A, L254A, H255A, R262A, Y355A and F359A, exhibited normal growth in the presence of 8 mM Fe, whereas wild-type AtNRAMP3 could not grow under the same conditions (Figure 3). These findings suggest that these 13 residues were required for the Fe transport activity of AtNRAMP3. In addition, six other mutations, including L71A, E77A, D79A, E126A, S256A and R379A, partially impaired the ability of AtNRAMP3 to confer Fe sensitivity (Figure 3). These mutants were able to grow in the presence of 8 mM Fe but could not grow as healthy as the empty vector. Finally, another 40 mutations behaved similar to wild-type (Figure S1).

### 2.4. Effects of Amino Acid Substitutions on Mn Transport Activity

All point mutations were tested for Mn transport activity. The expression of wild-type AtNRAMP3 increased Mn sensitivity of yeast strain *pmr1* in the presence of 0.6 mM Mn. Mutants G61A, P62A, D72A, N75A, E194A, L254A, H255A, R262A, Y355A and F359A did not increase Mn sensitivity under the same condition (Figure 3), suggesting that these amino acids were critical for Mn^2+^ transport activity. Seven mutations, L71A, E77A, D79A, E126A, H250A, S256A and R379A, showed decreased transport activity (Figure 3), indicating that these residues were involved in Mn transport activity. Additionally, other mutants retained nearly the same level of Mn transport activity as that of the wild-type (Figure S1).

### 2.5. Effects of Amino Acid Substitutions on Cd Sensitivity

All point mutations were also introduced into the Cd-hypersensitive yeast strain *ycf1* to test for Cd sensitivity. The wild-type AtNRAMP3 expressing *ycf1* conferred higher sensitivity to external Cd (Figure 3), however, several substitute mutants conferred reduced sensitivity, including G61A, P62A, L71A, D72A, E194A, H250A and H255A. At the same time, we did not find any mutants that abolished the Cd transport activity (Figure 3). Other mutations showed no change in Cd transport activity (Figure S1).

### 2.6. The Methionine Involved in Metal Substrate Selectivity

**Figure 4:**
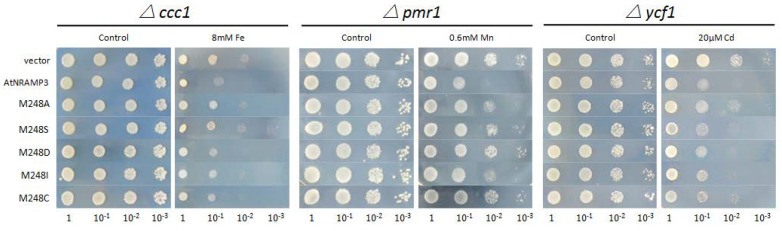
Met-248 mutations showed different phenotypes in yeast. Substitution of Met248 with Ala, Ser, Ile, Cys and Asp were transformed into *S. cerevisiae* mutants Δ*ccc1*, Δ*pmr1* and Δ*ycf1*, respectively. Transformants were pre-cultured in SD-Ura overnight. Pre-cultured cells were diluted to an OD_600_ of 1.0, 10 μL of each cell suspension was spotted onto selective SD-Ura plates with metal supplementation as indicated. The plates were incubated for 72 h at 30 °C.

A previous study proposed that the conserved Met in the metal-binding site stabilized metal substrates, and the ion were surrounded predominantly by harder ligands along with the soft sulfur of Met in the SLC11/NRAMP family [27]. To better understand the function of M248 in AtNRAMP3, this residue was extensively and variably substituted with Ala, Ile, Cys, Ser or Asp, respectively, and the metal selectivity of the mutated transporters was assessed in the yeast mutants. As shown in Figure 4, M248A showed a significant decrease in Cd transport activity and partially retained Fe and Mn transport. M248S abolished Fe and Mn transport but still transported Cd. Substitution of Met248 with Cys or Asp resulted in reductions in Fe and Cd transport activity but abolished Mn transport. M248I showed reductions in Fe, Mn, and Cd transport. These results indicated that M248 was involved in ion selectivity in AtNRAMP3.

### 2.7. The N-Terminus but Not the C-Terminus Is Essential to Transport Activity

To determine the possible roles of the N- and C-terminal regions of AtNRAMP3, several deletion mutants were constructed, followed by testing of their transport activity for Fe, Mn, and Cd. The results showed that there was no effect on the transport activity when the C-terminal 20 to 50 amino acids (including α-helix 12) were deleted (Figure 3). However, after 20–50 amino acids in the N-terminus were deleted, the truncated AtNRAMP3 abolished Fe and Mn transport activity, and reduced Cd transport activity (Figure 3). These findings indicated that the N-terminus was essential for the function of AtNRAMP3.

### 2.8. Metal Accumulation Assays

**Figure 5:**
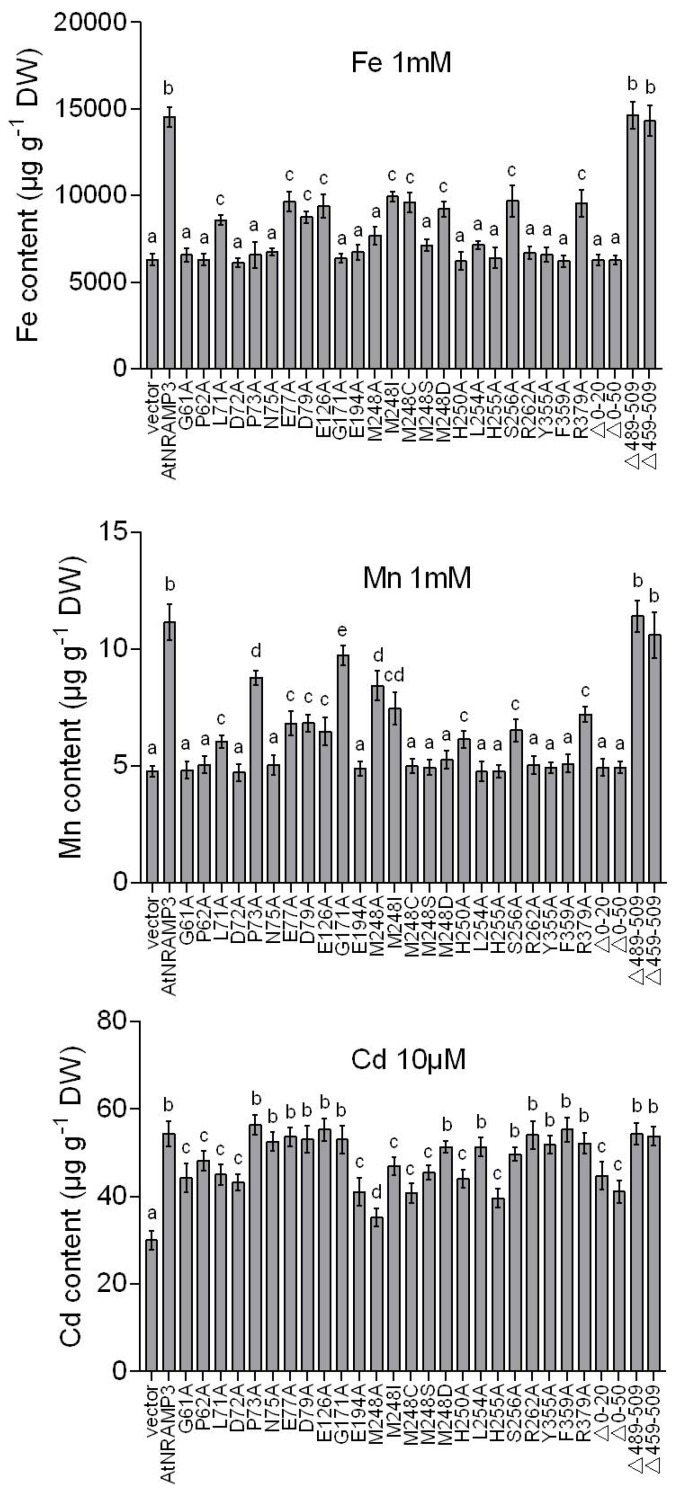
Concentration of Fe^2+^, Mn^2+^ and Cd^2+^ in yeast cells expressing wild-type AtNRAMP3 or mutated AtNRAMP3 variants. Metal accumulation in yeast cells were determined in the wild-type BY4741 (WT). BY4741 was transformed with wild-type AtNRAMP3 or mutated AtNRAMP3 variants, transformed cells were grown in liquid SD medium supplemented with 1 mM Fe^2+^, 1 mM Mn^2+^ or 10 μM Cd^2+^ at an initial OD600 = 0.1 for 24 h. Error bars indicate the standard deviations of three independent biological repeats. Means with different letters are significantly different (Tukey’s test, *p* < 0.05).

To further investigate the metal transport activity and selectivity of the AtNRAMP3 mutants, the accumulation of Fe, Mn and Cd were compared in wild-type yeast strains that expressed the AtNRAMP3 or AtNRAMP3 mutants. The results showed that the content of Fe, Mn, or Cd in AtNRAMP3 expressing cells had increased by 2-fold than those in the cells transformed with empty vector, consistent with a previous study [18]. C-terminus deletion has no effects on the transport activity, and most of the mutations impaired Fe and Mn transport. Furthermore, we identified several mutations that partially impaired Cd transport (Figure 5). The effected of the mutations on transport activity are summarized in Table 1. To further evaluate the effect of the mutations, we selected several representative mutants to test the growth curves in liquid culture (Figure S2) where results were consistent with the conclusions above.

**Figure 6:**
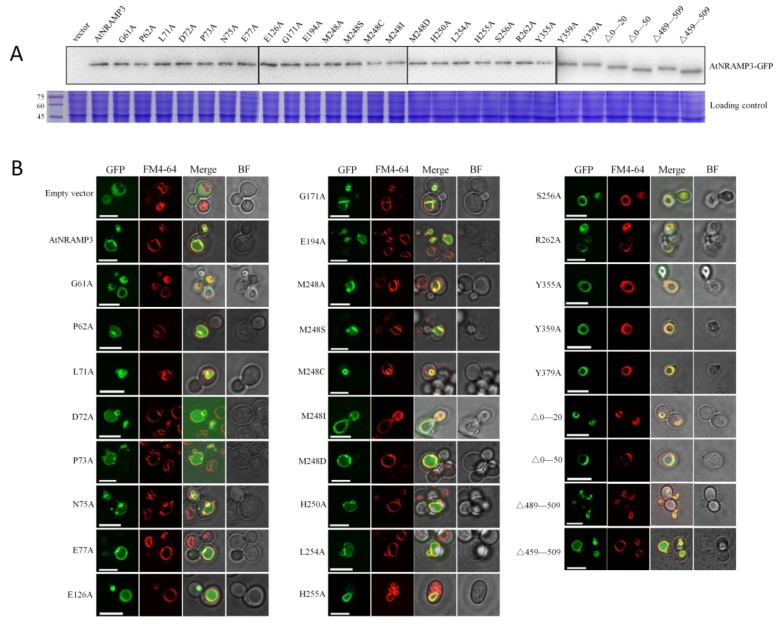
Expression and subcellular localization of AtNRAMP3 mutant proteins. (**A**) Western blot analysis of crude membrane fraction prepared from the BY4741 cells expressing wild-type and mutant variants of AtNRAMP3-GFP using an anti-GFP antibody. Sodium dodecyl sulfate polyacrylamide gel electrophoresis (SDS-PAGE) was used as a loading control. (**B**) C-terminal GFP fusion protein expressed in the *S. cerevisiae* strain BY4741. Cells were visualized 24 h after induction. From left to right, GFP fluorescence, FM4-64, merged images and bright-field. Scale bar: 5 μm.

To investigate whether the transport activity defects observed above were due to different protein levels between the wild-type AtNRAMP3 and mutants, we created GFP (green fluorescent protein)-fused protein constructs and transformed them into the wild-type yeast strain. The protein expression levels were checked by immunoblotting with anti-GFP antibody. The results showed that AtNRAMP3 and all mutant variants were expressed at similar level (Figure 6A). The subcellular localization of the mutants within the yeast cells showed a vacuolar localization, similar to that of the wild-type (Figure 6B). GFP-tagged AtNRAMP3 constructs also showed similar transport activity to the non-GFP tagged proteins (Figure S3).

### 2.9. Homologous Simulation of AtNRAMP3 Structure

The crystal structure of a member of the NRAMP family, EcoDMT, has been previously reported [27]. EcoDMT contains 12 transmembrane helices and a single ion-binding site. AtNRAMP3 shares an overall 30% identity with EcoDMT (Figure 2). To elucidate the role of mutated amino acid residues in AtNRAMP3 in transport activity and ion selectivity, we constructed a 3D homology model of AtNRAMP3 (Figure S4), based on the crystal structure of EcoDMT (PDB ID 5m8k), by using the SWISS-MODEL.

**Figure 7:**
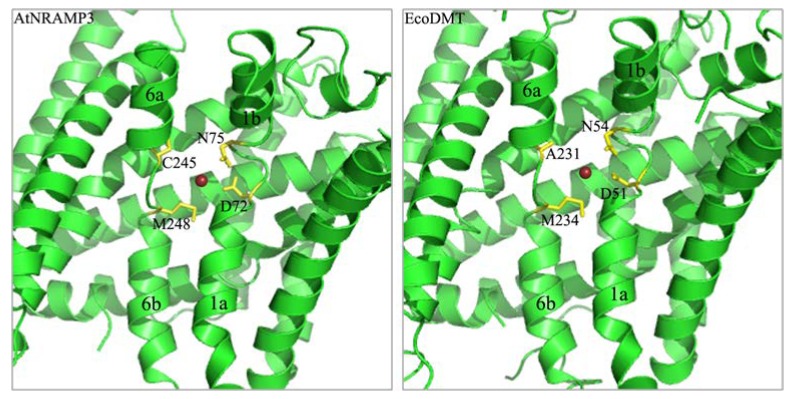
Putative ion-binding site of AtNRAMP3. Comparison of the putative ion-binding site of AtNRAMP3 (**left**) with characterized ion-binding sites of EcoDMT (**right**). The putative ion-binding site and homology model of AtNRAMP3 was based on the known ion-binding site and crystal structure of EcoDMT. The active ion-binding site of AtMRANP3 was conserved with ion-binding site of EcoDMT.

In EcoDMT, the active ion-binding site consisted of Asp51 and Asn54 from the nearby α-helix 1, Met234 in the unwound parts of α-helix 6, and the backbone carbonyl of Ala231 in α-helix 6a. In AtNRAMP3, these corresponded to D72, N75, M248 and C245 (Figure 7). Among these, Asp, Asn, and Met were strongly conserved within the NRAMP family (Figure 2).

**Figure 8:**
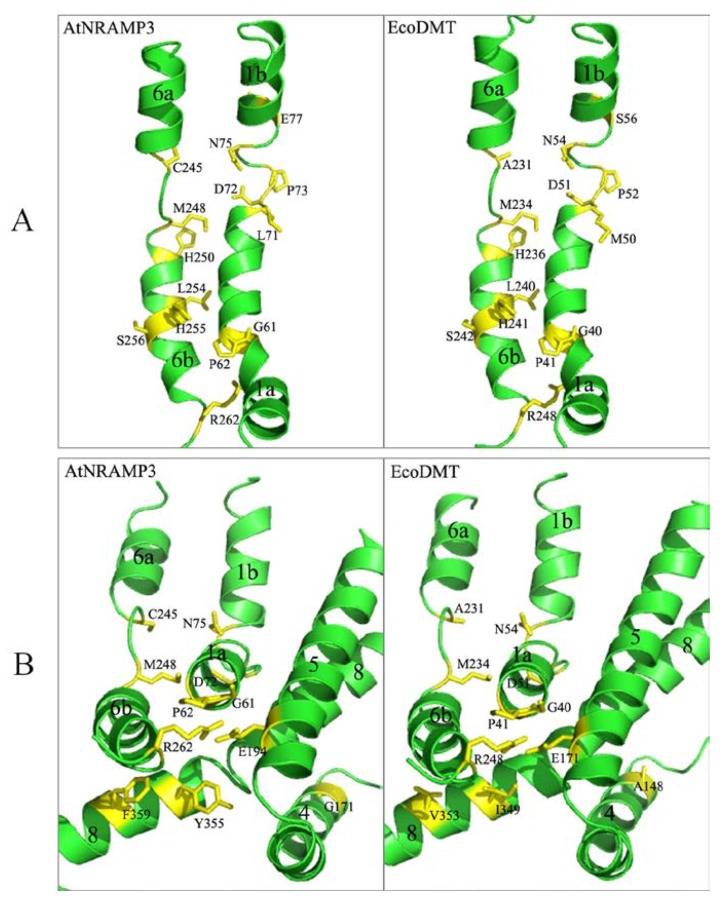
Residues with relevant roles in the transport activity of AtNRAMP3 (**left**) and the corresponding residues on the crystal structures of EcoDMT (**right**). (**A**) Residues located within α-helices 1 and 6 that affect transport activity are shown as sticks. (**B**) Some residues distal to the ion-binding site and necessary for transport activity are located on α-helices 4, 5 and 8.

Several mutations in AtNRAMP3 resulted in a significant reduction in Fe and Mn transport activity, including G61A, P62A, L71A, E77A, H250A, L254A, H255A, S256A and R262A. Furthermore, P73A resulted in a marked decrease in Fe transport activity but not Mn and Cd transport activity. These residues were highly conserved and located at the ion-binding site between α-helices 1 and 6 (Figure 8A). Other mutations that are located distant to the binding site, G171A, E194A, Y355A and F359A, also showed changes in transport activity. For example, G171A caused a reduction in Fe transport activity, whereas E194A, Y355A and F359A induced a significant decrease in Fe and Mn transport activity (Figure 8B).

## 3. Discussion

The structure and molecular transport mechanisms of the NRAMP proteins in bacteria have been studied; however, related information in plant NRAMP proteins remain poorly understood. This study aimed to identify the functional role of highly conserved residues in a plant NRAMP protein, AtNRAMP3. The ion-binding site of NRAMP was identified by the crystal structure that is formed by three residues (Asp, Asn and Met) that are strongly conserved from bacteria to humans and a backbone carbonyl group from a non-conserved residue [25,26,27]. In AtNRAMP3, these corresponded to D72, N75, M248, and the backbone carbonyl of C245 (Figure 2 and Figure 7). The mutations in ScaDMT (D49A, N52A), DraNRAMP (D56A), and hsDMT (D86A, N89A) were severely decreased Mn, Fe and Cd transport activity [26,27,28]. MntH scanning mutagenesis revealed that mutated the D34 and N37 were severely decreased Mn, Fe, Co, and Cd uptake [5,35,36,37]. In AtNRAMP3, the D72A and N75A mutations resulted in a significant reduction in Fe and Mn transport activity, however, D72A, but not N75A, showed a slight reduction in Cd transport activity. These results suggest that in AtNRAMP3, D72 and N75 are responsible for binding ions such as Fe and Mn, but nonspecific for Cd binding. The function of the conserved metal-binding amino acid Met has previously been investigated in a few species. In ScaDMT, the M226A mutation reduces Fe and Cd transport [26,28]. A parallel phenotype was observed in hsDMT, where the M265A mutation caused a significant impairment of Fe, Mn, and Cd transport activity [25,27]. In EcoDMT, the M-to-A, M-to-I and M-to-K mutations eliminated Mn transport. However, the M-to-A mutation did not affect Fe transport, similar to that reported in other bacterial homologs. In DraNRAMP, the M230A mutation did not alter Fe, Mn, and Co transport activity, but drastically decreased Cd transport and increased Mg and Ca transport [28]. These results suggest that Met at the ion-binding site in NRAMPs might be responsible for ion selectivity. In our study, we substituted Met248 with Ala, Ile, Cys, Ser and Asp, respectively. Interestingly, only M248A showed severely reduced Cd transport activity, which decreased Cd sensitivity while maintaining the ability of AtNRAMP3 to transport Mn, suggesting that short hydrophobic side chains such as –CH_3_ in Ala might not be conducive to Cd binding. This hypothesis will be further investigated in *Arabidopsis* sp. soon. Similarly, mutations in AtNRAMP4 that discriminate against Cd but do not affect the Fe transport have been identified [33]. These findings indicate that alterations in metal transport selectivity of transporters to discriminate against toxic metal substrates such as Cd, As and Pb have far-reaching significance.

TM1a movement is an integral part of Nramp conformational rearrangement and is required for metal transport [25,26,27]. The G45R or G45F mutant on the TM1a of DraNRAMP forms a steric wedge that prevents the protein from reaching the outward-open state, which in turn impairs Co and Fe uptake [25]. The mutation involving the highly conserved residue G61A in AtNRAMP3 also significantly impairs Fe and Mn transport, indicating the importance of this residue for proper conformational cycling for protein function.

The mutation G185R in humans and rodents impairs Fe uptake, which in turn causes anemia [29,30]. Bozzi et al. also observed a similar phenotype in DraNRAMP, indicating that G153R or similar substitutions likely alter the conformation of DraNRAMP, thereby changing the conserved metal-binding site [25]. The corresponding mutation G171A involving AtNRAMP3 TM4 selectively abrogates Fe transport activity while maintaining the ability to transport Mn and Cd. The amino acid G171 may thus be important to metal binding and selectivity in AtNRAMP3, similar to the findings of a previous report where NRAMP homolog mutations distant from the metal-binding site affect metal substrate selectivity [33].

The transport function of members of the NRAMP family is driven by the co-transport of H^+^, which provides energy for active transport and is crucial for the conformational changes of the protein [27,38,39]. Previous studies have shown that two histidines serve as H^+^ acceptors for the transport of protons, and mutations involving these two residues disrupt protein function [26,27,35,40]. In the present study, these two highly conserved histidines are H250 and H255, which agrees with the previous study, and mutations H250A and H255A in AtNRAMP3 severely impaired Fe and Mn transport and partially impaired Cd transport. The phenotype of these conserved residues suggests that plant NRAMP transporters may share a similar coupling mechanism.

Analysis of the crystal structures of NRAMP transporters has revealed an alternative access mechanism underlying conformational transitions from an outward-facing state to an inward-facing state [25,26,27]. In EcoDMT, the C-terminal parts of α-helix 4 and α-helix 5 are proximal to α-helix 1a and α-helix 6b when in the outward-facing state, and movements involving α-helices 1, 4, and 5 result in conformational changes into the inward-facing state; these conformations constantly circulate during transition metal ion transport [27]. Here, we found two residues (E194 from α-helix 5 and R262 from α-helix 6b) that play critical roles in Fe and Mn transport (Figure 3); these two residues are localized face-to-face between α-helix 5 and α-helix 6b in an outward-facing conformation (Figure 8B). These two residues may thus play an important role in stabilizing outward-facing conformation, which is essential to transport activity.

A previous study involving EcoDMT suggested that α-helix 12, which is located at the periphery of the protein, probably does not play a major role in transport [27]. The deletion of α-helix 12 in the C-terminal of AtNRAMP3 did not result in changes in transport activity (Figure 3). This result confirmed those earlier findings that α-helix 12 may not play a major role in transport activity. N-terminus deletions severely impaired Fe and Mn transport, but retained some Cd activity (Figure 3). The important role of the N-terminal the involved in ion selectivity and transport was also identified in AtMTP1 and TaHMA2 [41,42]. Part of that N-terminus conserved in plant NRAMP, the results suggested that the N-terminus may be essential for the function of plant NRAMP.

In conclusion, by using site-directed mutagenesis and metal toxicity growth assays in yeast and combined with 3D structure modeling based on the *E. coleocola* NRAMP family transporter EcoDMT, we identified residues in the plant NRAMP member AtNRAMP3 that play an important role in metal transport activity. The present study revealed the structure-function relationships of AtNRAMP3 in metal transport activity, which may potentially be applied to other plant NRAMP proteins.

## 4. Materials and Methods

### 4.1. Mutagenesis of AtNRAMP3

AtNRAMP3 was PCR amplified from the cDNA library and cloned into the *Not*I sites of pFL61 [43]. Site-directed mutagenesis was performed on pFL61-AtNRAMP3 using QuikChange II XL site-directed mutagenesis kits (Agilent Technologies, Foster, CA, USA) according to the manufacturer’s instructions. Sequences encoding the N- or C-terminal truncating mutants of AtNRAMP3 were PCR amplified and inserted into the same sites of the pFL61 vector. The induced mutations and primers used in this study are listed in Table S1. All mutations in this work were confirmed by DNA sequencing.

### 4.2. Yeast Transformation and Growth Analyses

The mutants and the wild-type yeast strains used in this study were obtained from the Euroscarf collection [44]. Yeast transformation was performed using the lithium acetate/PEG transformation method [45]. Positive colonies were selected on synthetic dropout (SD) plates containing synthetic defined medium without uracil (pH 6). Yeast strains expressing empty vector, wild-type AtNRAMP3, or mutated AtNRAMP3 variants were pre-cultured in SD-Ura liquid medium at 30 °C for 16 h. Pre-cultured cells were centrifuged and diluted into sterile water to an OD_600_ of 1.0, and 10-μL aliquots were spotted onto SD-Ura plates containing various metals at different concentrations. The plates were incubated at 30 °C for three days. The growth curve of yeast was measured based on the growth rate according to the OD_600_ values. Growth analyses were performed with three independent biological repeats, each of them replicated twice technically, and the representative data are shown.

### 4.3. Subcellular Localization in Yeast

For subcellular localization in the yeast, the WT and mutants were fused with mGFP and cloned into the yeast expression vector pFL61. The construct was transformed into strain BY4741. Yeast vacuolar membranes were selectively stained with the red lipophilic styryl dye FM4–64 (Invitrogen, Eugene, OR, USA) and GFP fluorescence was observed by confocal laser scanning microscopy (Confocal System-UitraView VOX, PerkinElmer, Waltham, MA, USA).

### 4.4. Metal Analysis

To measure metal accumulation, the wild-type yeast strain BY4741 was transformed with wild-type AtNRAMP3 or mutated AtNRAMP3 variants, and transformed cells were grown in liquid SD medium supplemented with 1 mM Fe^2+^, 1 mM Mn^2+^ or 10 μM Cd^2+^ at an initial OD_600_ = 0.1. After 24 h growth at 30 °C with shaking (220 rpm), cells were collected by centrifugation and washed three times with ice-cold 50 mM Tris-HCl (pH 6.5) and 10 mM EDTA and twice with water. Pellets were dried for 24 h at 70 °C and then digested in HNO_3_ at 120 °C for 45 min by using a microwave digester (UltraCLAVE IV, Milestone, Sorisole, Italy). Metal content was measured by ICP-MS (ELEMENT 2, Thermo Fisher Scientific, Waltham, MA, USA). The metal accumulation assays were performed with three independent biological repeats, each of them replicated twice technically.

### 4.5. Protein Extraction and Immunoblotting

Crude membrane fractions were extracted as previously described [46]. Protein extracts (10 μg) were loaded on 10% SDS-PAGE and transferred to a polyvinylidene fluoride membrane (Bio-Rad, Hercules, CA, USA). The membrane was probed with the anti-GFP antibody. After incubation with goat anti-mouse HRP conjugated IgG, target protein bands were revealed by enhanced chemiluminescence.

### 4.6. Bioinformatics Analysis of AtNRAMP3

AtNRAMP3 (GenBank Acc. No. AT2G23150) and its homologs were aligned using multalin [47]. The 3D model of AtNRAMP3 was generated by homology modeling using SWISSMODEL [48,49], based on the structure of EcoDMT (PDB ID 5m8k). Images were generated using PyMOL 1.6.x.

## Figures and Tables

**Table 1:** Summary of the mutants that affect the transport activity of AtNRAMP3. The transport activity of wild-type AtNRAMP3. −, none; + or ++, partial; +++, full.

Substitution	Fe Transport	Mn Transport	Cd Transport
AtNRAMP3	+++	+++	+++
G61A	−	−	++
P62A	−	−	++
L71A	+	+	++
D72A	−	−	++
P73A	−	++	+++
N75A	−	−	+++
E77A	+	+	+++
D79A	+	+	+++
E126A	+	+	+++
G171A	−	++	+++
E194A	−	−	++
M248A	−	++	+
M248S	−	−	++
M248D	+	−	++
M248I	+	+	++
M248C	+	−	++
H250A	−	+	++
L254A	−	−	+++
H255A	−	−	++
S256A	+	+	+++
R262A	−	−	+++
Y355A	−	−	+++
F359A	−	−	+++
R379A	+	+	+++
Δ0–20	−	−	++
Δ0–50	−	−	++
Δ489–509	+++	+++	+++
Δ459–509	+++	+++	+++

## Supplementary Materials

The following are available online at http://www.mdpi.com/1422-0067/19/5/1430/s1.

## Abbreviations

NRAMP	natural resistance-associated macrophage protein
TM	transmembrane
