# Supplementary material for: A Functional Study Identifying Critical Residues Involving Metal Transport Activity and Selectivity in Natural Resistance-Associated Macrophage Protein 3 in Arabidopsis thaliana
Source: Int J Mol Sci. 2018 May 10;19(5):1430. doi: 10.3390/ijms19051430 (PMC5983769; doi:10.3390/ijms19051430)
Supplement: Supplementary file 1 [file ijms-19-01430-s001.zip › Supplementary Files/Table S1.docx]

Table S1. List of primers used for cloning mutant AtNRAMP3 cDNAs, mutation sites are underlined.

|  | Sequence of forward primer | Sequence of reverse primer |
| --- | --- | --- |
| K54A  G61A  P62A  L65A  L71A  D72A  P73A  N75A  E77A  D79A  L93A  L111A  H118A  L119A  E121A  E126A  E140A  D147A  G171A  E194A  P229A  G241A  C245 | 5’- CACCACCGTTCTCATGGGCGAAGCTATGGTTATTC -3’  5’- CTATGGTTATTCACCGCACCTGGGTTTTTAATGAG -3’  5’- GGTTATTCACCGGAGCTGGGTTTTTAATGAGCATTG -3’  5’- ATTCACCGGACCTGGGTTTGCAATGAGCATTGCGT -3’  5’- ATGAGCATTGCGTTTGCAGATCCAGGGAATCTCG -3’  5’- GAGCATTGCGTTTTTAGCTCCAGGGAATCTCGAAG -3’  5’- GAGCATTGCGTTTTTAGCACCAGGGAATCTCGAAG -3’  5’- GTTTTTAGATCCAGGGGCTCTCGAAGGAGATCTTC -3’  5’- GATCCAGGGAATCTCGCAGGAGATCTTCAAGCCG -3’  5’- AGGGAATCTCGAAGGAGCTCTTCAAGCCGGTGCGG -3’  5’- GTACTCTTTGTTATGGGCTCTCATGTGGGCAACAG -3’  5’- GCTTTTGTCGGCTAGGGCTGGTGTTGCGACAGGTC -3’  5’- GTGTTGCGACAGGTCGTGCCTTAGCTGAGCTTTG -3’  5’- GCGACAGGTCGTCACGCAGCTGAGCTTTGTCGTG-3’  5’- CGTCACTTAGCTGCGCTTTGTCGTGATGAGTATC-3’  5’- CTGAGCTTTGTCGTGATGCGTATCCTACTTGGGC -3’  5’- GTTTTGTGGGTTATGGCTGCATTGGCTTTGATTG -3’  5’- GGCTTTGATTGGATCTGCTATTCAAGAAGTTATTG -3’  5’- GATTTTGCCTCTTTGGGCTGCTGTTGTTATTACTG -3’  5’- CGGAATAAGGAAGCTCGCGGCTGTGTTTGCAGTTC -3’  5’- CATTGGGATTTTGGTAGCGAAACTGAGTTCAAGAAC -3’  5’- CGATACAGAAAGCAGTTGCAGTTGTGGGTTGCAT -3’  5’-CAGTTGGAGTTGTGGGTGCCATTATAATGCCACAC-3’ | 5’- GAATAACCATAGCTTCGCCCATGAGAACGGTGGTG -3’  5’- CTCATTAAAAACCCAGGTGCGGTGAATAACCATAG -3’  5’- CAATGCTCATTAAAAACCCAGCTCCGGTGAATAACC -3’  5’- ACGCAATGCTCATTGCAAACCCAGGTCCGGTGAAT -3’  5’- CGAGATTCCCTGGATCTGCAAACGCAATGCTCAT -3’  5’- CTTCGAGATTCCCTGGAGCTAAAAACGCAATGCTC -3’  5’- CTTCGAGATTCCCTGGTGCTAAAAACGCAATGCTC - 3’  5’- GAAGATCTCCTTCGAGAGCCCCTGGATCTAAAAAC --3’  5’- CGGCTTGAAGATCTCCTGCGAGATTCCCTGGATC --3’  5’- CCGCACCGGCTTGAAGAGCTCCTTCGAGATTCCCT -3’  5’- CTGTTGCCCACATGAGAGCCCATAACAAAGAGTAC -3’  5’- GACCTGTCGCAACACCAGCCCTAGCCGACAAAAGC -3’  5’- CAAAGCTCAGCTAAGGCACGACCTGTCGCAACAC -3’  5’- CACGACAAAGCTCAGCTGCGTGACGACCTGTCGC -3’  5’- GATACTCATCACGACAAAGCGCAGCTAAGTGACG -3’  5’- GCCCAAGTAGGATACGCATCACGACAAAGCTCAG -3’  5’- CAATCAAAGCCAATGCAGCCATAACCCACAAAAC -3’  5’- CAATAACTTCTTGAATAGCAGATCCAATCAAAGCC -3’  5’- CAGTAATAACAACAGCAGCCCAAAGAGGCAAAATC -3’  5’- GAACTGCAAACACAGCCGCGAGCTTCCTTATTCCG -3’  5’- GTTCTTGAACTCAGTTTCGCTACCAAAATCCCAATG -3’  5’- ATGCAACCCACAACTGCAACTGCTTTCTGTATCG -3’  5’- GTGTGGCATTATAATGGCACCCACAACTCCAACTG -3’ |

|  | Sequence of forward primer | Sequence of reverse primer |
| --- | --- | --- |
| M248A  M248C  M248D  M248S  M248I  P249A  H250A  N251A  L254A  H255A  S256A  R262A  L342A  L343A  G346A  Q347A  S348A  S349A  T350A  I351A  T352A  G353A  T354A  Y355A  G357A | 5’-GTGGGTTGCATTATAGCGCCACACAATGTGTTTCT-3’  5’-GTGGGTTGCATTATAGCGCCACACAATGTGTTTCT-3’  5’- GTGGGTTGCATTATAGCGCCACACAATGTGTTTCT -3’  5’- GTGGGTTGCATTATAGCGCCACACAATGTGTTTCT -3’  5’- GTGGGTTGCATTATAGCGCCACACAATGTGTTTCT -3’  5’- TGGGTTGCATTATAATGGCACACAATGTGTTTCTTCAC -3’  5’- GTTGCATTATAATGCCAGCCAATGTGTTTCTTCAC -3’  5’- GCATTATAATGCCACACGCTGTGTTTCTTCACTCAG -3’  5’- GCCACACAATGTGTTTGCTCACTCAGCTCTTGTTC -3’  5’- CACAATGTGTTTCTTGCCTCAGCTCTTGTTCAATC -3’  5’- CAATGTGTTTCTTCACGCAGCTCTTGTTCAATCTAG -3’  5’- CAGCTCTTGTTCAATCTGCAGAAGTCGATAAACG -3’  5’- CATTTGGGCGATCGGGGCATTAGCTGCTGGCCAAAG -3’  5’- TGGGCGATCGGGCTAGCAGCTGCTGGCCAAAGCAG -3’  5’- CGGGCTATTAGCTGCTGCCCAAAGCAGCACTATTAC -3’  5’- GCTATTAGCTGCTGGCGCAAGCAGCACTATTACCG -3’  5’- ATTAGCTGCTGGCCAAGCCAGCACTATTACCGGTA -3’  5’- GCTGCTGGCCAAAGCGCCACTATTACCGGTACATA -3’  5’- GCTGGCCAAAGCAGCGCTATTACCGGTACATATGC -3’  5’- TGGCCAAAGCAGCACTGCTACCGGTACATATGCG -3’  5’- CAAAGCAGCACTATTGCCGGTACATATGCGGGACA -3’  5’- AGCAGCACTATTACCGCTACATATGCGGGACAGT -3’  5’- GCAGCACTATTACCGGTGCATATGCGGGACAGTTC -3’  5’- CACTATTACCGGTACAGCTGCGGGACAGTTCATAA -3’  5’- TACCGGTACATATGCGGCACAGTTCATAATGGGCG -3’ | 5’- AGAAACACATTGTGTGGCGCTATAATGCAACCCAC -3’  5’- AGAAACACATTGTGTGGCGCTATAATGCAACCCAC -3’  5’- AGAAACACATTGTGTGGCGCTATAATGCAACCCAC -3’  5’- AGAAACACATTGTGTGGCGCTATAATGCAACCCAC -3’  5’- AGAAACACATTGTGTGGCGCTATAATGCAACCCAC -3’  5’- GTGAAGAAACACATTGTGTGCCATTATAATGCAACCCA -3’  5’- GTGAAGAAACACATTGGCTGGCATTATAATGCAAC -3’  5’- CTGAGTGAAGAAACACAGCGTGTGGCATTATAATGC -3’  5’- GAACAAGAGCTGAGTGAGCAAACACATTGTGTGGC -3’  5’- GATTGAACAAGAGCTGAGGCAAGAAACACATTGTG -3’  5’- CTAGATTGAACAAGAGCTGCGTGAAGAAACACATTG -3’  5’- CGTTTATCGACTTCTGCAGATTGAACAAGAGCTG -3’  5’- CTTTGGCCAGCAGCTAATGCCCCGATCGCCCAAATG -3’  5’- CTGCTTTGGCCAGCAGCTGCTAGCCCGATCGCCCA -3’  5’- GTAATAGTGCTGCTTTGGGCAGCAGCTAATAGCCCG -3’  5’- CGGTAATAGTGCTGCTTGCGCCAGCAGCTAATAGC -3’  **5’-** TACCGGTAATAGTGCTGGCTTGGCCAGCAGCTAAT**-3’**  5’-TATGTACCGGTAATAGTGGCGCTTTGGCCAGCAGC-3’  5’-GCATATGTACCGGTAATAGCGCTGCTTTGGCCAGC-3’  5’-CGCATATGTACCGGTAGCAGTGCTGCTTTGGCCA-3’  5’-TGTCCCGCATATGTACCGGCAATAGTGCTGCTTTG-3’  5’-ACTGTCCCGCATATGTAGCGGTAATAGTGCTGCT-3’  5’-GAACTGTCCCGCATATGCACCGGTAATAGTGCTGC-3’  5’-TTATGAACTGTCCCGCAGCTGTACCGGTAATAGTG -3’  5’-CGCCCATTATGAACTGTGCCGCATATGTACCGGTA -3’ |

|  | Sequence of forward primer | Sequence of reverse primer |
| --- | --- | --- |
| Q358A  F359A  I360A  M361A  G362A  G363A  F364A  L365A  R374A  R379A  E405A  F417A  △0-20  △0-50  △489-509  △459-509 | 5’- CGGTACATATGCGGGAGCGTTCATAATGGGCGGGT -3’  5’- GTACATATGCGGGACAGGCCATAATGGGCGGGTTTC -3’  5’- CATATGCGGGACAGTTCGCAATGGGCGGGTTTCTTA -3’  5’- GCGGGACAGTTCATAGCGGGCGGGTTTCTTAATTTC -3’  5’- GGGACAGTTCATAATGGCCGGGTTTCTTAATTTC -3’  5’- CAGTTCATAATGGGCGCGTTTCTTAATTTCAAAAT -3’  5’- GTTCATAATGGGCGGGGCTCTTAATTTCAAAATGA -3’  5’- CATAATGGGCGGGTTTGCTAATTTCAAAATGAAGA -3’  5’- ATGAAGAAATGGTTGGCAGCTTTGATCACACGAAG -3’  5’- GAGAGCTTTGATCACAGCAAGCTGCGCTATCATTC -3’  5’- CTCGATGTCTTAAACGCGTGGCTTAACGTGCTTC -3’  5’- GTCCATTCAAATCCCCGCTGCACTCATTCCCTTAC -3’  5’- CAAATATAAAACCAGCGGCCGCATGGCGTACGATGAAACAGAGAAG -3’  5’- CAAATATAAAACCAGCGGCCGCATGTTCTCATGGAAGAAGCTATGGT -3’  5’- CAAATATAAAACCAGCGGCCGCATGCCACAACTCGAGAACAACG -3’  5’- CAAATATAAAACCAGCGGCCGCATGCCACAACTCGAGAACAACG -3’ | 5’-ACCCGCCCATTATGAACGCTCCCGCATATGTACCG -3’  5’-GAAACCCGCCCATTATGGCCTGTCCCGCATATGTAC -3’  5’-TAAGAAACCCGCCCATTGCGAACTGTCCCGCATATG -3’  5’-GAAATTAAGAAACCCGCCCGCTATGAACTGTCCCGC -3’  5’-GAAATTAAGAAACCCGGCCATTATGAACTGTCCC -3’  5’-ATTTTGAAATTAAGAAACGCGCCCATTATGAACTG -3’  5’-TCATTTTGAAATTAAGAGCCCCGCCCATTATGAAC-3’  5’-TCTTCATTTTGAAATTAGCAAACCCGCCCATTATG-3’  5’-CTTCGTGTGATCAAAGCTGCCAACCATTTCTTCAT-3’  5’-GAATGATAGCGCAGCTTGCTGTGATCAAAGCTCTC-3’  5’-GAAGCACGTTAAGCCACGCGTTTAAGACATCGAG-3’  5’-GTAAGGGAATGAGTGCAGCGGGGATTTGAATGGAC-3’  5’-GAAAAAAATTGATCGCGGCCGCTCAATGACTAGACTCCGCTTTGAAC -3’  5’-GAAAAAAATTGATCGCGGCCGCTCAATGACTAGACTCCGCTTTGAAC -3’  5’-GAAAAAAATTGATCGCGGCCGCTCAGAGGATGAATGCACCATAAGAAG-3’  5’- GAAAAAAATTGATCGCGGCCGCTCAAAGATAACCGTTGATCATTATC-3’ |
