# Supplementary figures and images for: A Functional Study Identifying Critical Residues Involving Metal Transport Activity and Selectivity in Natural Resistance-Associated Macrophage Protein 3 in Arabidopsis thaliana
Source: Int J Mol Sci. 2018 May 10;19(5):1430. doi: 10.3390/ijms19051430 (PMC5983769; doi:10.3390/ijms19051430)

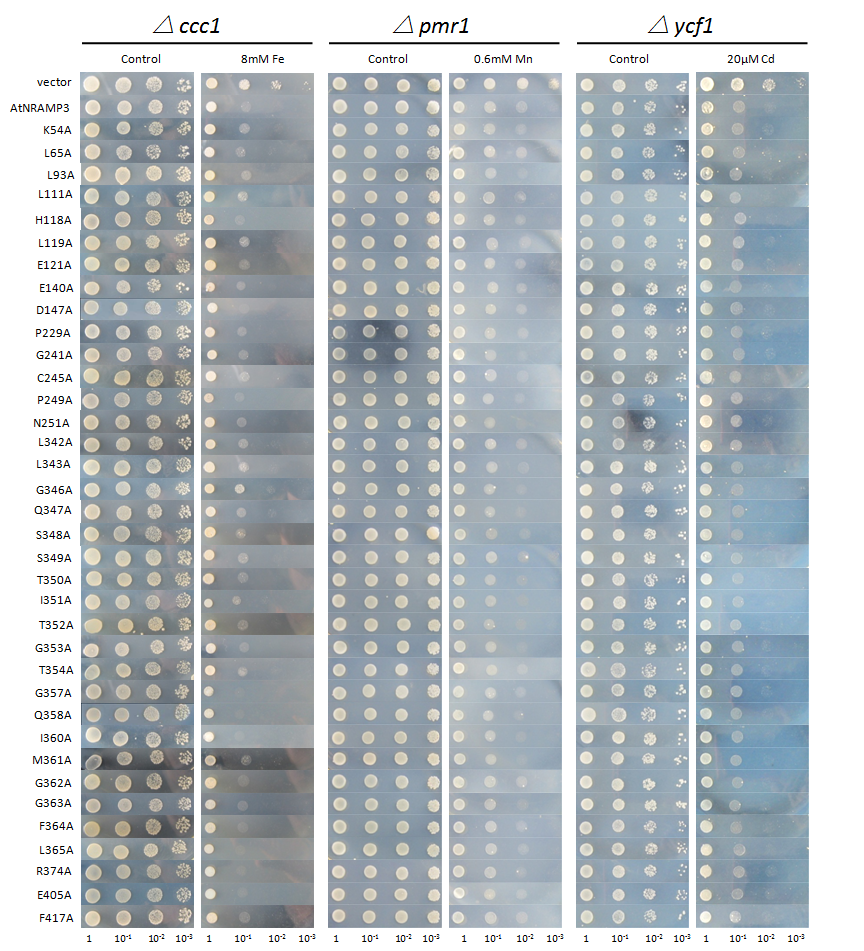

Supplement: Supplementary file 1 [file ijms-19-01430-s001.zip › Supplementary Files/Figure S1.tif]

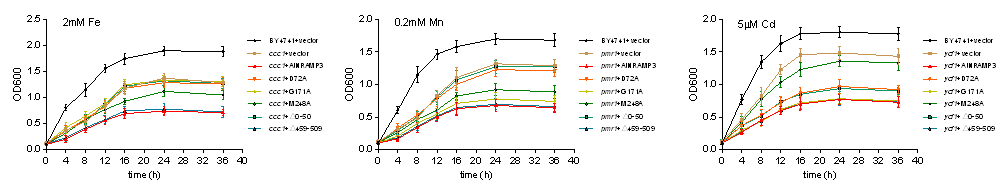

Supplement: Supplementary file 1 [file ijms-19-01430-s001.zip › Supplementary Files/Figure S2.tif]

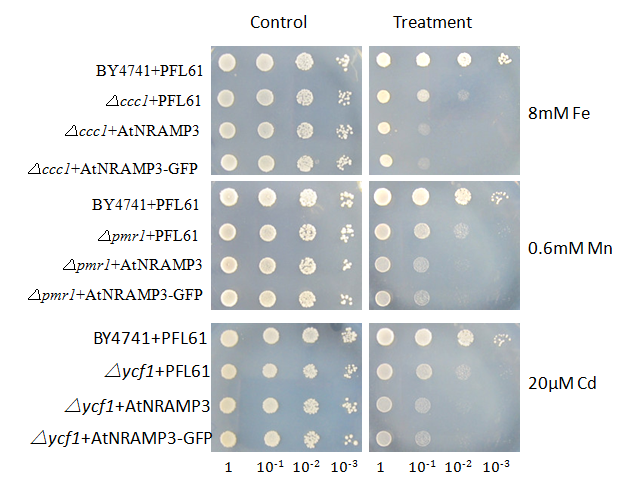

Supplement: Supplementary file 1 [file ijms-19-01430-s001.zip › Supplementary Files/Figure S3.tif]

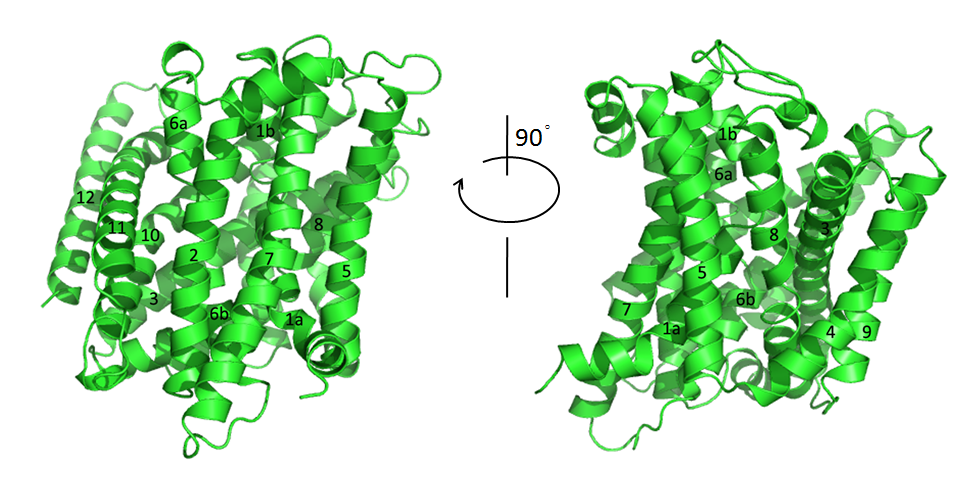

Supplement: Supplementary file 1 [file ijms-19-01430-s001.zip › Supplementary Files/Figure S4.tif]
